# Overexpression of FoxM1 predicts poor prognosis of intrahepatic cholangiocarcinoma

**DOI:** 10.18632/aging.101706

**Published:** 2018-12-21

**Authors:** Lingyun Liu, Jian Wu, Yu Guo, Wenxuan Xie, Bin Chen, Yi Zhang, Shaoqiang Li, Yunpeng Hua, Baogang Peng, Shunli Shen

**Affiliations:** 1 Department of Hepatic Surgery, The First Affiliated Hospital of Sun Yat-sen University, Guangzhou 510080, Guangdong, China; 2 Department of Hepatobiliary and Pancreatic Surgery, Affiliated Hospital of Guilin Medical University, Guilin 541000, Guangxi, China; 3 Department of General Surgery, The First Affiliated Hospital of Sun Yat-sen University, Guangzhou 510080, Guangdong, China; * Equal contribution

**Keywords:** FoxM1, intrahepatic cholangiocarcinoma, prognosis, biomarkers, cell proliferation, metastasis

## Abstract

FoxM1 is an oncoprotein that is significantly overexpressed in many malignancies including hepatocellular carcinoma, but its role in intrahepatic cholangiocarcinoma (ICC) remains unclear. This study explores the expression of FoxM1 in human ICC, its relationships with clinical outcomes, and its role in the proliferation, migration, and invasion of ICC *in vitro* and *in vivo*. The results show that FoxM1 was markedly elevated in tumor tissues *versus* the paired peritumoral tissues. Overexpression of FoxM1 was correlated with multiple tumor nodules, tumor size > 5 cm, positive lymph node metastasis and advanced TNM stage. Cox analysis revealed that overexpression of FoxM1 is an independent prognostic indicator for both the overall survival and disease-free survival of ICC patients after hepatectomy. Furthermore, up/downregulation of FoxM1 markedly promoted/inhibited ICC cell proliferation, migration, and invasion *in vitro* and *in vivo*. Bioinformatic analysis indicated that overexpression of FoxM1 resulted in the dysregulation of multiple signaling pathways in ICC, and selected components of some key signaling pathways such as c-Myc signaling were confirmed *in vitro*. In addition, overexpression of FoxM1 enhanced MMP-9 and MMP-2 protein expression in ICC cells. In conclusion, FoxM1 promotes ICC progression and is a reliable predictor of poor prognosis in ICC.

## Introduction

As a common hepatic malignancy derived from second-degree bile tracts, ICC constitutes 5-10% of human primary liver cancers [1]. The incidence of and mortality due to ICC has rapidly increased in recent decades, with geographic variations [2]. Due to the insidious onset and highly invasive biological behavior of ICC, patients with ICC often have advanced clinical stage at diagnosis and have missed the opportunity for radical surgery [3]. In addition, early tumor recurrence and metastasis is common in ICC patients after surgery [4]. Unfortunately, effective chemoradiotherapies and molecularly targeted therapies are unavailable. The current 3-year and 5-year overall survival rates of patients with ICC are only 31% and 18%, respectively [5]. Therefore, there is an urgent need to obtain a more detailed understanding of the molecular and pathobiological mechanisms of ICC.

As a member of the forkhead gene family, Forkhead Box Protein M1 (FoxM1) is an important transcription factor that regulates cell proliferation and apoptosis [6–8]. FoxM1 promotes tumorigenesis by enhancing cell proliferation and facilitates the invasion and metastasis of tumors [9–11], Several studies have found that FoxM1 is overexpressed and highly correlated with the clinical prognosis in patients with various solid tumors, including breast cancer [12], cervical cancer [13], gastric cancer [14], and non-small cell lung cancer [15]. The hepatitis B virus X protein induces the upregulation of FoxM1, which promotes the invasion and metastasis of hepatitis B virus-related HCC. A high expression level of FoxM1 is an independent adverse factor for recurrence and poor survival in those patients after hepatectomy [16]. Furthermore, FoxM1 affects chromosomal stability in HCC tissues and plays a catalytic role in the progression of HCC [17]. These findings indicated that FoxM1 plays an important role in the tumorigenesis, invasion and metastasis of multiple malignancies including HCC. Kazutaka et al. found abnormally increased levels of FoxM1 by analyzing the whole genome expression profiles of 25 ICC samples [18]. However, the role of FoxM1 in ICC has not been fully investigated before now.

In this study, we assessed the expression of FoxM1 in ICC. Then, we analyzed the correlation of FoxM1 with the clinicopathological characteristics and prognosis of 184 ICC patients after hepatectomy. Furthermore, we investigated the functional roles and underlying mechanisms of action of FoxM1 by which it regulates the proliferation, migration, and invasion of ICC cells *in vitro* and *in vivo*.

## RESULTS

### FoxM1 is upregulated in ICC

**Figure 1:**
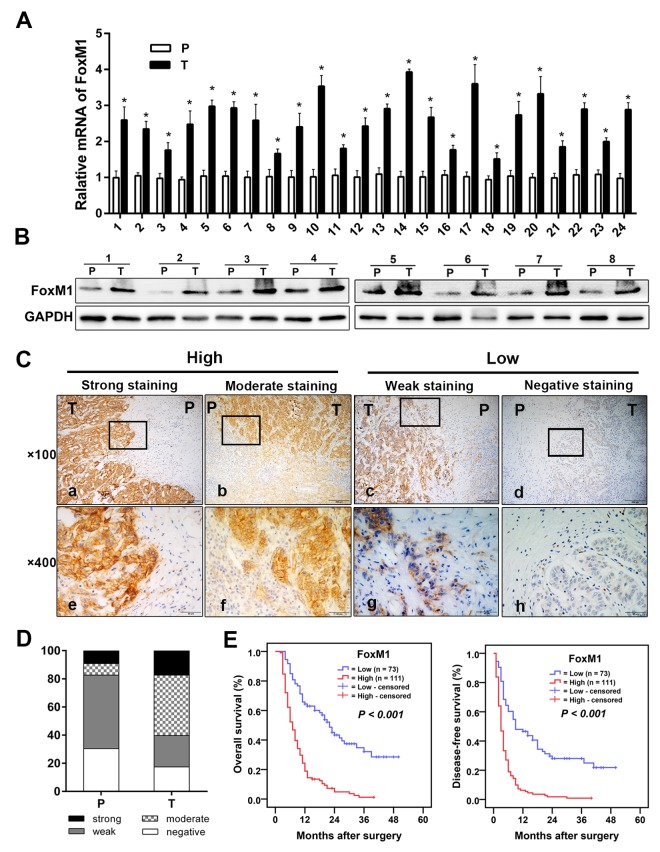
**FoxM1 was upregulation in ICC and correlated with survival. The mRNA expression** (**A**) and protein expression (**B**) of FoxM1 in ICC tumor tissues compared with the expression in paired peritumoral tissues. (**C)** Representative immunohistochemical staining images of FoxM1 in ICC. (**D**) The bar graph shows the statistics for the staining intensity of FoxM1 in 184 ICC tumor samples and paired peritumoral tissues. (**E**) Kaplan-Meier curves for the overall survival and disease-free survival of 184 patients with ICC according to the expression of FoxM1. Abbreviations: ICC, intrahepatic cholangiocarcinoma; P, peritumoral tissue; T, tumor tissue. ^*^P < 0.05.

qRT-PCR and Western blotting analysis showed that the mRNA and protein expression levels of FoxM1 were significantly higher in ICC cancer tissues than in the matched peritumoral tissues (Fig. 1A, B). Then, we used immunohistochemical staining to detect the protein expression of FoxM1 in 184 ICC samples. The results showed that FoxM1 was mainly expressed in the cell membrane and cytoplasm of tumor cells. Based on the results of immunohistochemical staining, the entire cohort of 184 ICC cases was dichotomized into the FoxM1^high^ expression group (> 4 points, n = 111) and the FoxM1^low^ expression group (≤ 4 points, n = 73) (Fig. 1C). The details of these patients were described in a previous study [19]. The clinicopathological features of all ICC patients are listed in Table 1.

**Table 1:** The relationships between FoxM1 and the clinicopathological variables of patients with ICC (n = 184).

**Category**	**Subcategory**	**No**	**FoxM1**		***χ^2^***	***r***	***P***
			**High** ***(n = 111)***	**Low** ***(n = 73)***			
Age (years)	≤ 60 > 60	117 67	69 (62.2%) 42 (37.8%)	48 (65.8%) 25 (34.2%)	0.245		0.620
Gender	Female Male	85 99	46 (41.4%) 65 (58.6%)	39 (53.4%) 34 (46.6%)	2.544		0.111
Cirrhosis	No Yes	138 46	82 (73.9%) 29 (26.1%)	56 (76.7%) 17 (23.3%)	0.189		0.664
HBsAg	Negative Positive	133 51	75 (67.6%) 36 (32.4%)	58 (79.5%) 15 (20.5%)	3.105		0.078
Tumor number	Single Multiple	121 63	60 (54.1%) 51 (45.9%)	61 (83.6%) 12 (16.4%)	**17.030**	**0.304**	**<0.001**
Tumor size (cm)	≤ 5 > 5	70 114	28 (25.2%) 83 (74.8%)	42 (57.5%) 31 (42.5%)	**19.503**	**0.326**	**<0.001**
Capsulation	No Yes	111 73	70 (63.1%) 41 (36.9%)	41 (56.2%) 32 (43.8%)	0.876		0.349
Differentiation^*^	W+M P	125 59	70 (63.1%) 41 (36.9%)	55 (75.3%) 18 (24.7%)	3.048		0.081
LNM	No Yes	111 73	55 (49.5%) 56 (50.5%)	56 (76.7%) 17 (23.3%)	**13.576**	**0.272**	**<0.001**
Vascular invasion	No Yes	173 11	105 (94.6%) 6 (5.4%)	68 (93.2%) 5 (6.8%)	0.163		0.686
TNM^†^	I+II III+IV	79 105	39 (35.1%) 72 (64.9%)	40 (54.8%) 33 (45.2%)	**6.947**	**0.194**	**0.008**
Resection margin	R0 R1	108 76	63 (56.8%) 48 (43.2%)	45 (61.6%) 28 (38.4%)	0.434		0.510
CEA (IU/L)	≤ 5.0 > 5.0	107 77	60 (54.1%) 51 (45.9%)	47 (64.4%) 26 (35.6%)	1.931		0.165
CA19-9 (IU/L)	≤ 35 > 35	65 119	38 (34.2%) 73 (65.8%)	27 (37.0%) 46 (63.0%)	0.146		0.702

Abbreviations: FoxM1, Forkhead Box Protein M1; ICC, intrahepatic cholangiocarcinoma; HBsAg, hepatitis B surface antigen; W+M, well + moderately differentiated; P, poorly differentiated; LNM, lymph node metastasis; TNM, tumor node metastasis; CEA, carcinoembryonic antigen; CA19-9, carbohydrate antigen 19-9.

*According to the World Health Organization (WHO) classification of tumors of the digestive system 2010.

† Based on the seventh edition of the cancer staging manual of the American Joint Committee on Cancer.

### Overexpression of FoxM1 is correlated with ICC progression and adverse prognosis

A higher expression level of FoxM1 was correlated with the presence of multiple tumors (χ^2^ = 17.030, *P* < 0.001), tumor diameter > 5 cm (χ^2^ = 19.503, *P* < 0.001), lymph node metastasis (χ^2^ = 13.576, *P* < 0.001), and advanced TNM stage (χ^2^ = 6.947, *P* = 0.008) (Table 1). However, there were no significant relationships between FoxM1 expression and clinicopathological parameters such as age, gender, cirrhosis, hepatitis B virus infection, tumor encapsulation, tumor differentiation, vascular invasion, resected margin, serum CEA, and serum CA19-9 (all *P* > 0.05, Table 1). The results suggested that overexpression of FoxM1 was markedly associated with adverse clinicopathological factors and may function as a biomarker of poor prognosis in patients with ICC.

The OS and DFS of the FoxM1^high^ expression group were significantly lower than those of the FoxM1^low^ expression group (all *P* < 0.001, Fig. 1E). The median OS of the FoxM1^high^ expression group was significantly lower than that of the FoxM1^low^ expression group (7 months *vs.* 22 months), and the median DFS of the FoxM1^high^ expression group was also markedly shorter than that of the FoxM1^low^ expression group (3 months *vs.* 9 months). In addition, the 1-, 3-, and 5-year OS rates of the FoxM1^high^ expression group were 18.9%, 1.2%, and 1.2%, respectively, which were significantly lower than those of the FoxM1^low^ expression group (64.4%, 32.1%, and 28.5%, respectively). The same trend was seen in the 1-, 3-, and 5-year DFS rates of the FoxM1^high^ and FoxM1^low^ expression groups (6.3%, 0.9%, and 0.9% *vs.* 46.6%, 28.1%, and 21.8%, respectively). The Cox regression proportional hazards model for multivariate analysis showed that tumor differentiation, resected margins, and the FoxM1 expression level were independent prognostic predictors for the OS of patients with ICC (all *P* < 0.05, Table 2). Tumor size, resected margin, and the FoxM1 expression level were independent prognostic indicators for DFS (all *P* < 0.05, Table 2). The above results indicated that high expression levels of FoxM1 were significantly correlated with ICC progression and adverse prognosis.

**Table 2:** Survival analysis for prognostic factors of patients with ICC (n = 184).

**Category**	**Overall Survival (OS)**				**Disease-Free Survival (DFS)**		
	**Univariate** ***P***	**Multivariate**			**Univariate** ***P***	**Multivariate**	
		**HR (95% CI)**	***P***			**HR (95% CI)**	***p***
Age (> 60 vs. ≤ 60 years)	0.723		NA		0.437		NA
Cirrhosis (No vs. Yes)	0.303		NA		0.268		NA
HBsAg (No vs. Yes)	0.916		NA		0.465		NA
Child-Pugh stage (A vs. B)	0.272		NA		0.148		NA
Tumor size (cm) (≤ 5 vs. > 5)	**0.006**		NS		**< 0.001**	**1.631 (1.147-2.319)**	**0.007**
Number (Single vs. Multiple)	**0.003**		NS		**0.001**		NS
Differentiation (W+M vs. P)	**0.002**	**1.544 (1.099-2.169)**	**0.012**		**0.020**		NS
Capsulation (No vs. Yes)	0.206		NA		0.647		NA
Vascular invasion (No vs. Yes)	0.112		NA		0.114		NA
TNM (I+II vs. III+IV)	**0.002**		NS		**0.003**		NS
Resection margin (R0 vs. R1)	**0.001**	**0.656 (0.474-0.908)**	**0.011**		**0.001**	**0.704 (0.514-0.966)**	**0.029**
LNM (No vs. Yes)	**0.003**		NS		**0.011**		NS
CEA (ng/mL) (≤ 5 vs. > 5)	**0.008**		NS		**0.001**		NS
CA19-9 (IU/mL) (≤ 35 vs. > 35)	0.324		NA		0.554		NA
FoxM1 (High vs. Low)	**< 0.001**	**3.275 (2.270-4.723)**	**< 0.001**		**< 0.001**	**2.653 (1.850-3.806)**	**< 0.001**

Abbreviations: ICC, intrahepatic cholangiocarcinoma; HBsAg, hepatitis B surface antigen; W+M, well + moderately differentiated; P, poorly differentiated; TNM, tumor node metastasis; LNM, lymph node metastasis; CEA, carcinoembryonic antigen; CA19-9, carbohydrate antigen 19-9; FoxM1, Forkhead Box Protein M1; NA, not applicable; NS, not significant.

### FoxM1 promoted the proliferation, migration, and invasion of ICC *in vitro*

**Figure 2:**
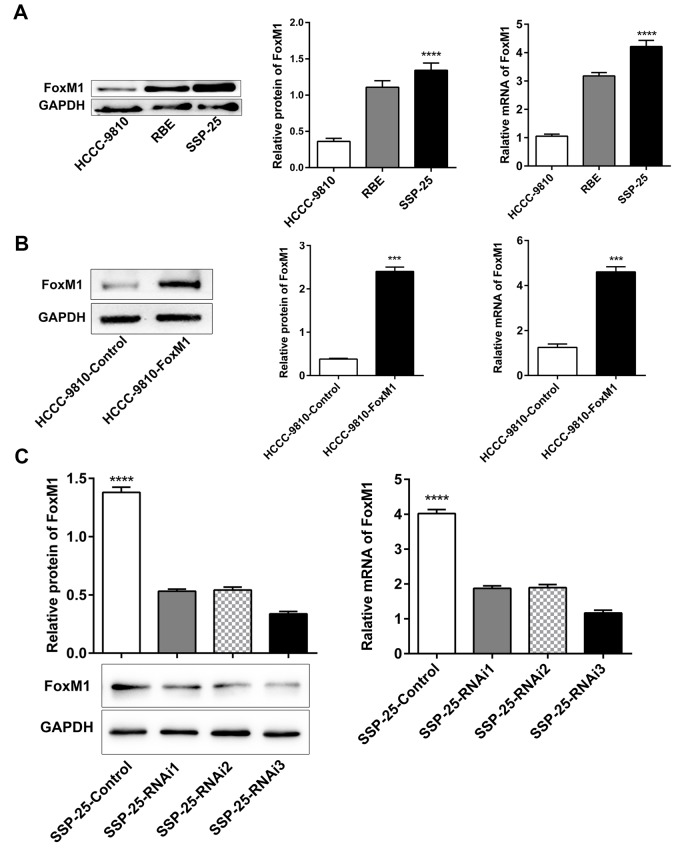
**Selection and establishment of stably transfected ICC cell lines with FoxM1.** (**A**) The protein and mRNA expression of FoxM1 in ICC cell lines (HCCC-9810, RBE, and SSP-25). Western blotting and qRT-PCR analysis showed successful overexpression (**B**) and knockdown (**C**) of FoxM1 in ICC cells (HCCC-9810 and SSP-25, respectively). ^***^P < 0.001, ^****^P < 0.0001.

To verify the exact biological effect of FoxM1 on ICC, we first established cell lines that stably up/downregulated FoxM1 expression. The FoxM1 mRNA and protein expression in 3 ICC cell lines were respectively detected by qRT-PCR and Western blotting. It was found that the expression of FoxM1 was highest in the SSP-25 cell line and lowest in HCCC-9810 (*P* < 0.0001, Fig. 2A). Thus, HCCC-9810 was selected for targeted upregulation of FoxM1 (HCCC-9810-FoxM1, compared with its control HCCC-9810-Control), and SSP-25 was selected for targeted downregulation of FoxM1 (SSP-25-shFoxM1, compared with its control SSP-25-Control) by lentiviral transfection. The transfection efficiencies of both cell lines reached greater than 90%, and the cells were further screened using RPMI-1640 mediums supplemented with puromycin reagent (1μg/mL). The results showed that the transfected cells (HCCC-9810-FoxM1, SSP-25-shFoxM1) could stably up/downregulate the protein and mRNA expression of FoxM1 compared with their respective control cells (all *P* < 0.001, Fig. 2B, C). In addition, both cell lines were able to consistently express the labeled green fluorescence and grew well. Therefore, the selected cell lines were used for further formal experiments.

**Figure 3:**
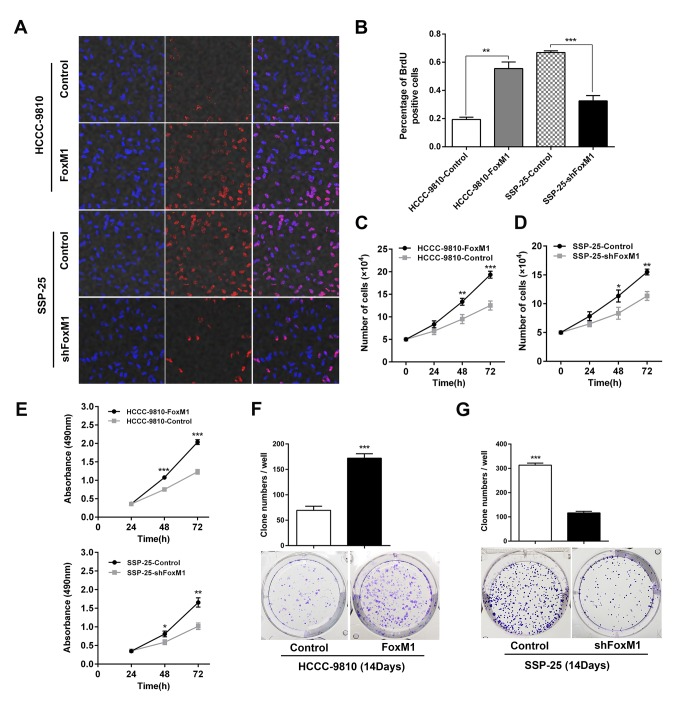
**FoxM1 promoted the proliferation of ICC *in vitro*.** The proliferative abilities of the indicated ICC cells were determined by Brdu incorporation assay (**A**, **B**), and cell number counting (**C**, **D**), MTT assays (**E**) and plate cloning tests (**F**, **G**, statistics are shown with a bar graph). ^*^P < 0.05, ^**^P < 0.01, ^***^P < 0.001.

**Figure 4:**
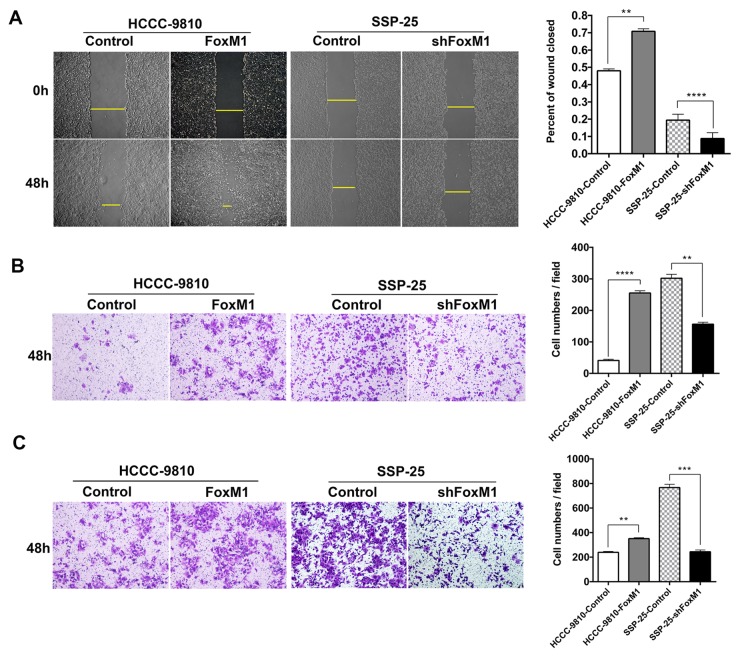
**FoxM1 promoted the migration and invasion of ICC cells *in vitro*.** The motility of ICC cells was detected by scratch assays (**A**) and Transwell migration assays (**B**). The invasiveness of ICC cells was determined by Transwell invasion assays (**C**). Statistics are shown with a bar graph. ^**^P < 0.01, ^***^P < 0.001, ^****^P < 0.0001.

Next, BrdU incorporation assay, MTT assays and plate cloning experiments were performed to detect the effect of FoxM1 on ICC cell proliferation. The results showed that the cell line with upregulated FoxM1 (HCCC-9810-FoxM1) had a significantly higher proliferative capacity than its control cell line (HCCC-9810-Control) (all *P* < 0.01, Fig. 3A-C, E, and F), while the proliferative ability of the cell line with downregulated FoxM1 (SSP-25-shFoxM1) was markedly lower than that of its control cell line (SSP-25-Control) (all *P* < 0.05, Fig. 3A, B, D, E, and G).In addition, it was indicated that plating efficiency of HCCC-9810-Control and HCCC-9810-FoxM1 were 13.8% (69/500, cells/well) and 34.4% (172/500, cells/well), while SSP-25-Control and SSP-25-shFoxM1 were 62.6% (313/500, cells/well) and 23.2% (116/500, cells/well) respectively (all shown with mean from at least three independent assays). Furthermore, the cell scratch assay was used to compare the horizontal migration ability of cells with up- and downregulated FoxM1. The results revealed that 48 hours after the scratch wound was inflicted, the percentage of the scratch repaired in the FoxM1 overexpression group (HCCC-9810-FoxM1) was significantly higher than that in the control group (HCCC-9810-Control) (*P* < 0.01, Fig. 4A), while the percentage of the wound closed in the FoxM1 downregulated group (SSP-25-shFoxM1) was markedly lower than that of its control group (SSP-25-Control) (*P* < 0.0001, Fig. 4A). We further used Transwell migration assays to compare the vertical motility of the transfected cells. The results showed that 48 hours after the start of the experiment, the number of penetrating cells in the HCCC-9810-FoxM1 group was notably greater than that of the HCCC-9810-Control group (*P* < 0.0001, Fig. 4B), while the number of penetrating cells in the SSP-25-shFoxM1 group was significantly lower than that in the SSP-25-Control group (*P* < 0.01, Fig. 4B). This tendency also existed in the subsequent Transwell invasion assay. The number of cells on the lower surface of the membrane in the HCCC-9810-FoxM1 group was markedly greater than that in the HCCC-9810-Control group (*P* < 0.01, Fig. 4C), and the number of penetrating cells in the SSP-25-shFoxM1 group was significantly lower than that of the SSP-25-Control group (*P* < 0.001, Fig. 4C). Together, the above results indicated that FoxM1 enhanced the proliferative, migrative and invasive capacities of ICC cells *in vitro*.

### FoxM1 promoted ICC progression *in vivo*

**Figure 5:**
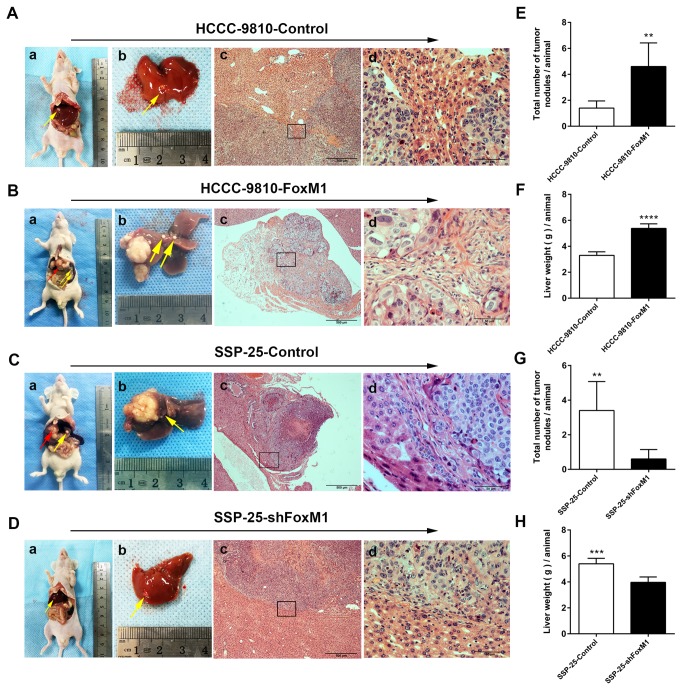
**Overexpression of FoxM1 promoted ICC progression *in vivo*.** Representative images of nude mouse ICC hepatic xenograft models (red arrow head show primary xenografted tumor, while the yellow arrow indicates metastasis in (**B-**a**, B-**b and **C-**a**, C-**b) and HE-stained sections (**A**-c, **B**-c, **C**-c, and **D**-c: magnification × 40; **A**-d, **B**-d, **C**-d, and **D**-d: magnification × 400). The total number of tumor nodules/animal (**E**, **G**) and liver weight (g)/animal (**F**, **H**) of the hepatic xenograft tumors showed marked differences between the experimental and control groups (n = 5 per group). Statistics are shown with a bar graph. ^**^P < 0.01, ^***^P < 0.001, ^****^P < 0.0001.

We then examined the effects of FoxM1 on ICC progression *in vivo* with a nude mouse liver tumorigenicity model (Fig. 5A, B, C, and D). The number of HCCC-9810-FoxM1-derived tumor nodules (including metastasis) was significantly greater than that in the HCCC-9810-Control group (*P* < 0.01, Fig. 5E). In contrast, the number of SSP-25-shFoxM1-derived tumor nodules (including metastasis) was markedly lower than that in the SSP-25-Control group (*P* < 0.01, Fig. 5G). Similarly, the weight of the liver including the HCCC-9810-FoxM1-derived tumors was significantly heavier than that of its control group (*P* < 0.0001, Fig. 5F), while the weight of liver including SSP-25-shFoxM1-derived tumors was markedly lighter than that of its control group (*P* < 0.001, Fig. 5H). These results show that FoxM1 promotes ICC progression *in vivo*.

### Overexpression of FoxM1 upregulates the activity of the c-Myc signaling pathway

Currently, bioinformatics is a powerful tool for data mining in the microarray RNA database. In this study, GSEA analysis was performed to find potential differentially expressed genes and the related signaling pathways based on the high/low expression of FoxM1 mRNA in ICC. The results of the GSEA gene signaling pathway enrichment analysis are shown in Table 3, including the c-Myc, P53, WNT canonical, c-MET, and ATM pathways, which are involved in the proliferation, invasion, metastasis, and apoptosis of ICC. The results indicated that FoxM1 may play a wide range of roles in different disease stages of ICC.

**Table 3:** Enrichment analysis of the effect of FoxM1 expression on related signaling pathways in ICC.

**No.**	**Signaling pathways**	**ES**	**NES**	**Nom P-value**	**FDR q-value**
1	PID_MYC_PATHWAY	0.583	1.705	0.016	0.089
2	PID_MYC_ACTIV_PATHWAY	0.601	1.973	0.000	0.009
3	PID_ATM_PATHWAY	0.635	1.871	0.001	0.026
4	PID_ATR_PATHWAY	0.745	2.121	0.000	0.001
5	PID_MET_PATHWAY	0.463	1.464	0.098	0.225
6	KEGG_P53_SIGNALING_PATHWAY	0.612	2.079	0.000	0.003
7	PID_P53_REGULATION_PATHWAY	0.545	1.728	0.004	0.077
8	PID_P53_DOWNSTREAM_PATHWAY	0.472	1.744	0.003	0.071
9	PID_WNT_CANONICAL_PATHWAY	0.574	1.547	0.050	0.172
10	REACTOME_SIGNALING_BY_WNT	0.528	1.555	0.081	0.169
11	REACTOME_INTRINSIC_PATHWAY_FOR_APOPTOSIS	0.537	1.643	0.020	0.129
12	PID_TELOMERASE_PATHWAY	0.476	1.539	0.051	0.170
13	PID_TAP63_PATHWAY	0.471	1.568	0.031	0.165
14	PID_PLK1_PATHWAY	0.791	2.076	0.000	0.003
15	PID_P38_MK2_PATHWAY	0.550	1.524	0.040	0.178
16	PID_FOXO_PATHWAY	0.532	1.561	0.047	0.167
17	PID_BARD1_PATHWAY	0.759	1.983	0.000	0.009

Abbreviations: ES, enrichment Score; NES, normalized enrichment score; Nom P-value, nominal p-value; FDR q-value, false discovery rate q-value. FDR q-value < 0.250 was considered different.

**Figure 6:**
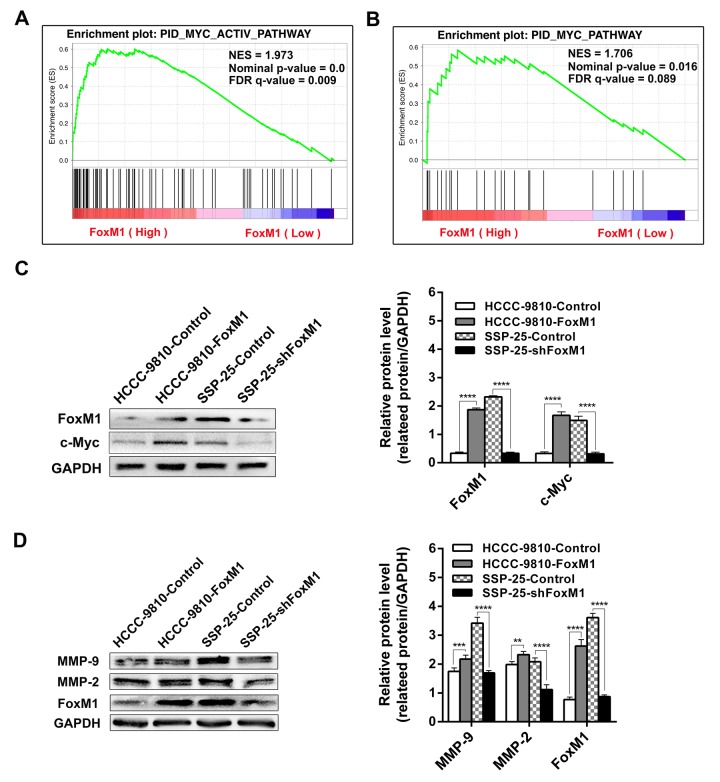
**GSEA (Gene Set Enrichment Analysis) of the c-Myc signaling pathway based on the high and low expression of FoxM1 in ICC.** The results indicated marked correlations of FoxM1 expression in ICC with the c-Myc signaling pathway (**A** and **B**). Subsequently, Western blotting verified that the overexpression of FoxM1 upregulates the activity of c-Myc signaling (**C**) and promotes MMP-9 and MMP-2 (**D**) protein expression in ICC. Statistics are shown with a bar graph. ^**^P < 0.01, ^***^P < 0.001, ^****^P < 0.0001.

The GSEA analysis showed that the MYC_ACTIV_PATHWAY (Fig. 6A) and the MYC_PATHWAY (Fig. 6B) had higher enrichment values, and both FDR q-values were < 0.250 when FoxM1 was highly expressed, which indicated that the expression levels of FoxM1 affect the activity of the MYC_PATHWAY. Western blotting then confirmed that the expression levels of the c-Myc protein in HCCC-9810-FoxM1 were significantly higher than those in HCCC-9810-Control, while expression levels of the c-Myc protein in SSP-25-shFoxM1 were markedly lower than those in SSP-25-Control (all *P* < 0.0001, Fig. 6C). Those results showed that the overexpression of FoxM1 in ICC upregulates the activity of the c-Myc signaling pathway.

### Overexpression of FoxM1 promotes MMP-9 and MMP-2 protein expression in ICC

Furthermore, to investigate the underlying mechanisms by which FoxM1 promotes ICC invasion and metastasis, Western blotting was used to detect the expression of the MMP-9 and MMP-2 proteins in the transfected cells. The results showed that the expression levels of the MMP-9 and MMP-2 proteins in HCCC-9810-FoxM1 were significantly higher than those in HCCC-9810-Control, while the expression levels of the MMP-9 and MMP-2 proteins in SSP-25-shFoxM1 were markedly lower than those in SSP-25-Control (all *P* < 0.01, Fig. 6D). This result indicated that high expression levels of FoxM1 in ICC cells enhance MMP-9 and MMP-2 protein expression; FoxM1 may promote the invasion and metastasis of ICC cells by regulating the expression of the MMP-9 and MMP-2 proteins.

## DISCUSSION

Currently, ICC is still a treatment-refractory malignancy with a poor clinical outcome, even if the patient undergoes radical hepatectomy. Due to the poor understanding of the pathogenesis of ICC, there are no effective systemic treatments, including chemotherapies and molecularly targeted therapies [20]. Hence, it is of great importance to carry out further research to explore the underlying mechanisms of ICC tumorigenesis and progression. In the present study, we assessed the roles and possible mechanisms of action of FoxM1 in ICC.

The human FoxM1 gene, once named HFH-11 [21] and MPP-2 [22], is located in the centromeric region of chromosome 12p13.3; it is approximately 25 kb in length and includes 10 exons. Previous reports indicated that high expression levels of FoxM1 in multiple malignancies are associated with poor prognosis [23–25]. Recently, researchers have found that FoxM1 expression is increased in human HCC and is closely related to the clinicopathological features of HCC; overexpression of FoxM1 in HCC tumor tissues is an independent prognostic factor for poor OS and DFS in HCC patients. Therefore, FoxM1 is considered a prognostic biomarker for HCC and may be used as a target for HCC therapy [26]. The results of this study show that FoxM1 is highly expressed and is strongly related to poor prognosis and adverse pathological factors in ICC such as multiple tumors, tumor diameter > 5 cm, positive lymph node metastasis, and advanced TNM stage. In addition, patients with ICC who overexpress FoxM1 might have larger tumor burdens and earlier postoperative recurrence or metastasis. Subsequently, our data confirmed that patients with ICC with high expression levels of FoxM1 have worse clinical outcomes than those with low expression levels of FoxM1. Multivariate analysis verified that overexpression of FoxM1 is an independent adverse indicator for both OS and DFS in patients with ICC. Thus, we propose that FoxM1 is a candidate tumor promotor for the risk stratification and treatment of ICC.

Previous studies have shown that FoxM1 can act as an oncogenic protein by interacting with other proteins such as β-catenin or SMAD3, leading to the activation of the oncogenic WNT and TGF-β signaling pathways, respectively [27]. The expression of FoxM1 was significantly increased in MHCCLM3 HCC cells with high metastatic potential compared to SMMC7721 cells with low metastatic potential, and FoxM1 expression was closely related to the migration and invasion of HCC cells, thus promoting HCC metastasis [28]. In view of the overexpression of FoxM1 in ICC tissues, the downregulation of FoxM1 in adjacent peritumoral tissues and the negative correlation of the expression of FoxM1 with the prognosis of patients with ICC, we speculate that FoxM1 may play a role as an oncoprotein in the development of ICC. Indeed, in concordance with the abovementioned reports, our study showed that upregulation of the expression of FoxM1 increased the proliferation, migration, and invasion of ICC cells *in vitro* and *in vivo*, while downregulation of FoxM1 expression had the opposite effect. Therefore, we conclude that FoxM1 functions as an oncoprotein in the tumorigenesis and progression of ICC.

The interaction of FoxM1 with numerous signaling pathways plays an important role in the development of multiple solid tumors. FoxM1 is essential in the progression of Ras signaling-driven liver cancer [29]. Another study found that FoxM1 is a key downstream effector that regulates the MET signaling pathway, resulting in DNA damage in gastric cancer cells [30]. Similarly, the biological effects of FoxM1 are regulated by many signaling pathways. Researchers have found that the mitogen-activated protein kinase (MAPK) pathway regulates FoxM1 phosphorylation and controls its subcellular localization and transcriptional activity [31]. In addition, as a downstream component of the WNT signaling pathway, FoxM1 is critical for the transcriptional function of β-catenin in tumor cells [32]. Another report suggested that hepatitis B virus X protein enhances FoxM1 expression through the ERK/CREB signaling pathway, thereby promoting the invasion and metastasis of hepatitis B virus-related HCC cells [16].

The tumorigenesis and development of ICC are accompanied by abnormal changes in multiple cell signaling pathways, including the EGFR, c-MET, WNT, and AKT/PI3K signaling pathways [33]. Current studies have found that the activation of c-Myc promotes the proliferation and invasion of ICC cells [33]. Another study suggested that the simultaneous activation of AKT and N-Ras signals can rapidly promote hepatocarcinogenesis in mice through the FoxM1 and c-Myc pathways [34]. Our study detected that FoxM1 promotes ICC proliferation, migration, and invasion. In addition, the bioinformatics analysis found that the expression of FoxM1 in ICC was significantly associated with the c-Myc signaling pathway. Subsequently, Western blotting confirmed that upregulation of FoxM1 expression significantly increased c-Myc protein expression, whereas downregulation of FoxM1 markedly inhibited c-Myc protein expression. The results indicated that FoxM1 enhances the activity of the c-Myc signaling pathway in ICC cells and that FoxM1 may play a role in ICC tumorigenesis and disease progression through this pathway.

Matrix metalloproteinases are members of the zinc-calcium-dependent endopeptidase family and play an important role in tumor metastasis and angiogenesis [35]. Its members MMP-9 and MMP-2 are closely related to tumor metastasis [36]. Studies have found that downregulation of FoxM1 in pancreatic cancer cells reduced the expression of MMP-2 and MMP-9, thereby inhibiting pancreatic cancer cell migration and invasion [37]. Another study showed that FoxM1 promoted the invasion and disease progression of malignant glioma by enhancing MMP-2 gene transcription [38]. Specific a-disintegrin and metalloproteinase (ADAM) are overexpressed in many human cancers and related with poor clinical outcomes and tumor progression, silencing FoxM1 led to an inhibition of cell proliferation, tumor growth induced by ADAM-17 in hilar cholangiocarcinoma [39]. Our study found that FoxM1 overexpression enhanced ICC cell migration and invasion and correspondingly increased the expression of the MMP-9 and MMP-2 proteins. Therefore, we speculate that that upregulation of the expression of the MMP-9 and MMP-2 proteins may play a role in the promotion of ICC migration and invasion by FoxM1. However, whether the expression of MMP-9 and MMP-2 is regulated by FoxM1 directly or indirectly through the c-Myc signaling pathway is not yet clear.

In summary, the findings from this study indicate that FoxM1 is significantly upregulated in ICC, and its overexpression is markedly associated with tumor progression and poor clinical outcomes in patients with ICC after hepatectomy. Overexpression of FoxM1 promotes the proliferation, migration, and invasion of ICC cells. In addition, FoxM1 may play a key role in the pathogenesis and disease progression of ICC via upregulating and activating the c-Myc signaling pathway and facilitating the expression of the MMP-9 and MMP-2 proteins. As such, there are good reasons to believe that FoxM1 functions as an oncoprotein and is both a reliable indicator of prognosis and a promising novel therapeutic target in ICC.

## MATERIALS AND METHODS

### Clinical ICC samples

In this study, 184 patients diagnosed for the first time with ICC who underwent hepatectomy at the First Affiliated Hospital of Sun Yat-sen University in Guangzhou from April 2004 to September 2015 were consecutively selected. All cases were pathologically diagnosed as ICC. All patients were older than 18 years old and had complete clinical follow-up and pathological data. No other anticancer treatment measures were performed before surgery, including radiochemotherapy, immunotherapy, and percutaneous ablation. Paired paraneoplastic tissues were defined as intrahepatic tissues with no infiltrating tumor cells within 2 cm of the ICC tumor tissues. The degree of tumor differentiation was determined based on the 2010 version of the World Health Organization (WHO) classification of tumors of the digestive system [40]. The TNM stage was assigned according to the seventh edition of the American Joint Committee on Cancer (AJCC) Cancer Staging Manual [41]. This study was approved by the ethics committee of Sun Yat-sen University, and all patients provided written consent. Routine postoperative follow-up was performed with all patients. The disease-free survival (DFS) was defined as the period from the day of surgery to the time of tumor relapse, and the overall survival (OS) was defined as the period from the day of the surgery to the time of ICC-related death. Data were censored for patients without recurrence or cancer-related death at the time of the last follow-up (August 2016), as previously reported [19].

Archived paraffin-embedded tumors and paired peritumoral tissues derived from all 184 patients with ICC were used for immunohistochemistry. Another 24 paired samples from patients with ICC were randomly collected between October 2015 and September 2016 for the analysis of FoxM1 mRNA expression, from which 8 tumors and the paired peritumoral tissues were also used for the analysis of the protein expression levels of FoxM1 (details for those patients are listed in Supplementary Table S1).

### Quantitative Real-Time PCR (qRT-PCR)

Following the manufacturer's instructions, total RNA was extracted using TRIzol reagent (Invitrogen, USA) from the tissue samples frozen in liquid nitrogen or from the ICC cell lines. cDNA was derived from RNA by reverse transcription using the PrimeScript^TM^ RT Master Mix (Takara, Japan). Subsequently, the qRT-PCR analysis was performed using SYBR^®^Premix Ex Taq^TM^ II (Takara, Japan) on the Bio-Rad platform. The sequences of the primers used for human FoxM1 were 5’-CGTCGGCCACTGATTCTCAAA-3’ (forward) and 5’-GGCAGGGGATCTCTTAGGTTC-3’ (reverse). The relative mRNA expression was assessed by comparative cycle threshold methods and normalized to the internal control GAPDH.

### Western blotting

Western blotting was performed as described previously [42]. Briefly, the proteins extracted from the tissues or cells were subjected to SDS-PAGE and then transferred onto polyvinylidene difluoride (PVDF) membranes (Millipore, USA). The PVDF membranes were blocked with 5% bovine serum albumin (BSA) for 1 h at room temperature and then incubated with the corresponding primary antibodies overnight at 4˚C. GAPDH was used as an internal loading control. Subsequently, the PVDF membranes were washed adequately with TBST and incubated with the corresponding secondary antibodies. Finally, the PVDF membranes were detected by enhanced chemiluminescence solution (Millipore, USA). The primary antibodies used for Western blotting are listed in Supplementary Table S2.

### Immunohistochemistry (IHC)

Rabbit polyclonal anti-human FoxM1 antibodies (1:100, Abcam, USA) were used for IHC according to the manufacturer's instructions. Antigen heat retrieval was performed with 0.01 mol/L sodium citrate buffer (pH 6.0) before beginning the IHC staining protocol. The specific procedures for the IHC were performed as previously described [43]. Five random fields under the microscope for each slice were used for IHC scoring, with the mean as the final result. The results of IHC staining were judged by two investigators independently based on the following criteria: 1) The extent of positivity according to the proportion of positively stained cells as follows: 0, no positive staining cells; 1, 0-25%; 2, 26-50%; 3, 51-75%; and 4, > 75%; 2) Staining intensity was scored as follows: 0, negative staining; 1, weak staining (light yellow); 2, moderate staining (yellow-brown); 3, strong staining (brown); and 3) The immunoreactivity scores (IRSs) were determined by adding the above scores, yielding a range from 0 to 7. Samples with an IRS > 4 and those with an IRS ≤ 4 were dichotomized as the high and low expression groups, respectively.

### Cell lines and lentiviral transfection

Three human ICC cell lines, HCCC-9810, RBE and SSP-25, were purchased from the Cell Bank of Chinese Academy of Sciences (Shanghai, China). The cells were cultured in RPMI-1640 medium (Gibco, USA) contained 10% fetal bovine serum (FBS) (Gibco, USA), 100 U/ml penicillin (Gibco, USA), and 100 μg/ml streptomycin (Gibco, USA) in a humidified incubator at 37°C with 5% CO_2_. The short hairpin FoxM1 lentiviral vectors (GeneChem Co., Ltd. Shanghai, China) designed to downregulate the expression of FoxM1 were transfected into SSP-25 cells, which were then designated SSP-25-shFoxM1. The lentiviral vectors with FoxM1 were transfected into HCCC-9810 cells, which were then designated HCCC-9810-FoxM1. Meanwhile, the empty lentiviral vectors were transfected into SSP-25 and HCCC-9810 cells as controls, which were designated SSP-25-control and HCCC-9810-control, respectively. The lentiviral vectors carried puromycin resistance and green fluorescent sequences. Subsequently, puromycin (1 µg/ml) was used for screening the stable cell lines. The transfection procedure was performed based strictly on the manufacturer’s instructions. The short hairpin RNA (shRNA) and cDNA clone sequences are listed in Supplementary Table S3.

### Cell counting and Bromodeoxyuridine (BrdU) incorporation and immunofluorescence assay for cell proliferation

BrdU analysis were performed according to described previously [44]. For proliferation assay, cells were placed in a 6-well plate at a concentration of 5×10^4^ cells per well. After incubation for 24–72 hours, triplicate wells were harvested and cells were counted with a hemocytometer after trypan blue staining. For Brdu incorporation assay, cells were plated in 12-well plate. 48 hours later, cells were incubated with BrdU at final concentration of 10uM for 4hrs. Cells were then fixed by 4% paraformaldehyde at RT for 10mins, followed by 0.1% Triton X-100 permeabilization and 1.5N HCl DNA hydrolysis. Cells were then probed by O/N incubation with BrdU antibody (1:100, Abcam, ab221240). Samples were then subjected to DAPI nuclear counterstain. BrdU positive cells were then quantified and data were presented as percentage of BrdU positive cells.

### MTT [3-(4,5-Dimethyl-2-thiazolyl)-2,5-diphenyl-2H-tetrazolium bromide] assays

MTT assays were performed to investigate the cell proliferative ability of HCCC-9810-FoxM1 and SSP-25-shFoxM1 compared with their respective controls. These four cell lines were added to 96-well plates at a concentration of 2 × 10^3^ cells/well and were incubated for 24 hours, 48 hours, and 72 hours in an incubator with 37°C and 5% CO_2_. Then, the MTT solution (20 µl) (KeyGEN BioTECH, Nanjing, China) was added to each well at the indicated time point and further incubated for 4 h. Dimethyl sulfoxide reagent (150 µl) was added to replace with original culture fluid, after which the plates were shaken at 37°C for 10 minutes. The absorbance at 490 nm was measured to detect the number of living cells in each well.

### Plate colony-forming assays

The effects of FoxM1 up/downregulation on cell proliferation *in vitro* were also investigated by plate colony-forming assays. HCCC-9810-FoxM1, HCCC-9810- Control, SSP-25-shFoxM1 and SSP-25-Control in the logarithmic phase were digested by trypsin, counted, seeded into six-well plates at 500 cells/well, and then incubated for 14 days under conventional cell culture conditions. After three washes with PBS, the clone points were fixed with methanol at room temperature and then stained with 0.1% crystal violet. Subsequently, the cell colonies were carefully washed again, photographed, and counted.

### Scratch, transwell migration and invasion assays

Scratch, Transwell migration and invasion assays were used to detect the motility and invasiveness of the 4 tumor cell lines. The scratch experiment was performed to evaluate the horizontal motor ability of tumor cells. Cells in the logarithmic phase were grown to confluence in 6-well plates, and a wound line was scratched into the cell monolayer using a sterile 200 µL pipette tip. The detached cells were removed by washing 2~3 times with sterile PBS, which was then replaced by the serum-free RPMI-1640 medium. The percent wound closure was observed by a Leica inverted microscope at 0 and 48 hours after the scratch was generated.

In addition, to assess the vertical motion capability and invasiveness of tumor cells, Transwell migration and invasion assays were performed, respectively. A total of 5 × 10^4^ cells suspended in 50 μl of serum-free RPMI-1640 medium was added to the upper chamber of the insert with/without Matrigel (Transwell migration/invasion assays, respectively), and 600 μl of RPMI-1640 containing 10% FBS was placed into the lower chamber. After a 48-h incubation at 37°C and 5% CO_2_, the cells on the lower surface of the insert were counted after fixation in 4% paraformaldehyde and stained with 0.1% crystal violet for 30 min. The number of cells in 9 random microscopic fields (magnification, × 100) of each Transwell chamber were counted and photographed. All results are presented as the mean ± standard error (SE) of 3 independent experiments conducted in triplicate.

### Animal studies and HE staining

The animal studies were approved by the Ethics Committee of the First Affiliated Hospital of Sun Yat-sen University (Guangzhou, China) (Grant number: 2014-12). Male athymic BALB/C nude mice (4 weeks old) were purchased from the model animal research center of Nanjing University (Nanjing, China). Tumor cells (1×10^7^) in the logarithmic phase that were suspended in 200 μL of serum-free RPMI-1640 medium and Matrigel (BD Biosciences, USA; dilution, 1:1) were subcutaneously implanted into the flanks of the mice (2 mice in each group). Three weeks later, the xenografts were observed in all treated nude mice, with the largest diameter ranging from 0.5 cm to 1.2 cm. Then, those mice were sacrificed, and the xenografts were excised cut into pieces (1 × 1 × 1 mm^3^ per piece). The remaining nude mice (4 weeks old, n = 5 per group) were anesthetized by intraperitoneal injection of 2% sodium pentobarbital (45 mg/kg). A transverse upper abdominal incision into the abdominal cavity was used to expose the liver, and then a tumor mass was implanted in the largest hepatic lobe. All surgical operations were carried out on a sterile, clean bench in the SPF operating room and were completed within 2 hours without antibiotics. Eight weeks after the operation, all nude mice were euthanized and examined by exploratory surgery. The liver and lungs were quickly removed, and the livers were weighed and photographed. HE staining was performed using paraffin-embedded sections derived from 10% formalin-fixed tissue.

### Bioinformatics analysis (Gene Set Enrichment Analysis, GSEA)

The mRNA data of 36 patients with ICC were downloaded from The Cancer Genome Atlas (TCGA) (https://cancergenome.nih.gov/) database. The 36 ICC samples were divided into a high expression group and a low expression group according to the median total mRNA expression of FoxM1. Subsequently, a bioinformatics analysis was performed by the GSEA software (version 3.0) to find the differentially expressed genes and related signaling pathways derived from the high/low expression of FoxM1 in ICC [45,46]. In the GSEA analysis, we used the Molecular Signature Database (MsigDB) provided by the GSEA as the signaling pathways annotation source, as it contains common pathways annotation data from KEGG and REACTOME. Significantly enriched signaling pathways were screened through a permutation of 1000 cycles.

### Statistical analysis

The statistical analysis were performed with SPSS version 20.0 software for Windows (IBM, Chicago, IL, USA). Data are presented as the mean ± SE from at least three independent assays. Numerical variables were compared using Student's t-test or one-way ANOVA. The χ^2^ test or Fisher's exact test were performed to evaluate the differences in categorical data, and the correlations among variables were compared by Pearson’s bivariate correlate analysis. The survival differences were assessed using the Kaplan-Meier method with a log-rank test. The Cox regression proportional hazards model (backward stepwise) was performed to determine the independent prognostic predictors. *P* < 0.05 (two-tailed) was considered statistically significant.

### Ethics approval

This study was approved by the ethics committee of Sun Yat-sen University, and all patients provided written consent. All animal procedures were performed according to national guidelines and approved by the Ethics Committee of the First Affiliated Hospital of Sun Yat-sen University (Guangzhou, China) (Grant number: 2014-12).

## Supplementary Material

Supplementary Tables
