# Supplementary material for: Overexpression of FoxM1 predicts poor prognosis of intrahepatic cholangiocarcinoma
Source: Aging (Albany NY). 2018 Dec 21;10(12):4120–40. doi: 10.18632/aging.101706 (PMC6326658; doi:10.18632/aging.101706)
Supplement: Supplementary Tables [file aging-10-101706-s001.pdf]

SUPPLEMENTARY TABLES

Supplementary Table S1. Clinicopathologic data of 24 ICC patients for qRT-PCR and Western Blotting (n = 24)

Category	Subcategory	No.
Age (years)	≤60	15
	>60	9
Gender	Female	10
	Male	14
Liver cirrhosis	No	10
	Yes	14
Tumor number	Single	18
	Multiple	6
Tumor size (cm)	≤5	18
	>5	6
Differentiation *	W+M	17
	P	7
LNM	No	14
	Yes	10
Vascular invasion	No	21
	Yes	3
TNM †	I+II	8
	III+IV	16
CA19-9 (IU/L)	≤35	7
	>35	17
CEA (IU/L)	≤5.0	17
	>5.0	7

Abbreviations: ICC, Intrahepatic cholangiocarcinoma; W+M, well+moderated differentiation; P, poor differentiation; LNM, Lymph node metastasis; TNM, Tumor Node Metastasis; CA19-9, Carbohydrate antigen 19-9; CEA, Carcinoembryonic antigen.

* According to the World Health Organization (WHO) classification of tumors of the digestive system 2010;

† Based on seventh edition cancer staging manual of American Joint Committee on Cancer.

Supplementary Table S2. Primary Antibodies for WB and IHC.

Antibody	Concentration for WB	Concentration for IHC	Specificity	Company
FoxM1	1:1000	1:100	Rabbit	Abcam
GAPDH	1:2000	/	Rabbit	Bioss
c-Myc	1:1000	/	Rabbit	CST
MMP-9	1:1000	/	Rabbit	Proteintech
MMP-2	1:1000	/	Rabbit	Proteintech

Abbreviations: WB, Western Blotting; IHC, immunohistochemistry; CST, Cell Signaling Technology.

Supplementary Table S3. The sequence of shRNA and cDNA clone of FoxM1.

Name	Sequence
FoxM1-RNAi-1	CCAACAGGAGTCTAATCAA
FoxM1-RNAi-2	GCTGGGATCAAGATTATTA
FoxM1-RNAi-3	GGCCACCCTACTCTTACAT
ORF nucleotide sequence of FoxM1 (transcript variant 2)	CTAAATTCTGGCCGTTTTTGGCTTTTTTGTTAGACGAAGCTTGGG<br>CTGCAGGTCGACTCTAGAGGATCCCCGGGTACCGGTCGCCACCA<br>TGAAAACTAGCCCCCGTCGGCCACTGATTCTCAAAAGACGGAGG<br>CTGCCCCTTCCTGTTCAAAATGCCCCAAGTGAAACATCAGAGGA<br>GGAACCTAAGAGATCCCCTGCCCAACAGGAGTCTAATCAAGCAG<br>AGGCCTCCAAGGAAGTGGCAGAGTCCAACTCTTGCAAGTTTCCA<br>GCTGGGATCAAGATTATTAACCACCCCACCATGCCCAACACGCA<br>AGTAGTGGCCATCCCCAACAATGCTAATATTCACAGCATCATCA<br>CAGCACTGACTGCCAAGGGAAAAGAGAGTGGCAGTAGTGGGCC<br>CAACAAATTCATCCTCATCAGCTGTGGGGGAGCCCCAACTCAGC<br>CTCCAGGACTCCGGCCTCAAACCCAAACCAGCTATGATGCCAAA<br>AGGACAGAAGTGACCCTGGAGACCTTGGGACCAAAACCTGCAG<br>CTAGGGATGTGAATCTTCCTAGACCACCTGGAGCCCTTTGCGAG<br>CAGAAACGGGAGACCTGTGCAGATGGTGAGGCAGCAGGCTGCA<br>CTATCAACAATAGCCTATCCAACATCCAGTGGCTTCGAAAGATG<br>AGTTCTGATGGACTGGGCTCCCGCAGCATCAAGCAAGAGATGGA<br>GGAAAAGGAGAATTGTCACCTGGAGCAGCGACAGGTTAAGGTT<br>GAGGAGCCTTCGAGACCATCAGCGTCCTGGCAGAACTCTGTGTC<br>TGAGCGGCCACCCTACTCTTACATGGCCATGATACAATTCGCCAT<br>CAACAGCACTGAGAGGAAGCGCATGACTTTGAAAGACATCTATA<br>CGTGGATTGAGGACCACTTTCCCTACTTTAAGCACATTGCCAAGC<br>CAGGCTGGAAGAACTCCATCCGCCACAACCTTTCCCTGCACGAC<br>ATGTTTGTCCGGGAGACGTCTGCCAATGGCAAGGTCTCCTTCTGG<br>ACCATTCACCCCAGTGCCAACCGCTACTTGACATTGGACCAGGT<br>GTTTAAGCCACTGGACCCAGGGTCTCCACAATTGCCCGAGCACT<br>TGGAATCACAGCAGAAACGACCGAATCCAGAGCTCCGCCGGAA<br>CATGACCATCAAAACCGAACTCCCCCTGGGCGCACGGCGGAAGA<br>TGAAGCCACTGCTACCACGGGTCAGCTCATACCTGGTACCTATCC<br>AGTTCCCGGTGAACCAGTCACTGGTGTTGCAGCCCTCGGTGAAG<br>GTGCCATTGCCCCTGGCGGCTTCCCTCATGAGCTCAGAGCTTGCC<br>CGCCATAGCAAGCGAGTCCGCATTGCCCCCAAGGTGCTGCTAGC<br>TGAGGAGGGGATAGCTCCTCTTTCTTCTGCAGGACCAGGGAAAG<br>AGGAGAAACTCCTGTTTGGAGAAGGGTTTTCTCCTTTGCTTCCAG<br>TTCAGACTATCAAGGAGGAAGAAATCCAGCCTGGGGAGGAAAT<br>GCCACACTTAGCGAGACCCATCAAAGTGGAGAGCCCTCCCTTGG<br>AAGAGTGGCCCTCCCCGGCCCCATCTTTCAAAGAGGAATCATCT<br>CACTCCTGGGAGGATTCGTCCCAATCTCCCACCCCAAGACCCAA<br>GAAGTCCTACAGTGGGCTTAGGTCCCCAACCCGGTGTGTCTCGG<br>AAATGCTTGTGATTCAACACAGGGAGAGGAGGGAGAGGAGCCG<br>GTCTCGGAGGAAACAGCATCTACTGCCTCCCTGTGTGGATGAGC<br>CGGAGCTGCTCTTCTCAGAGGGGCCCAGTACTTCCCGCTGGGCC

GCAGAGCTCCCGTTCCCAGCAGACTCCTCTGACCCTGCCTCCCAG
CTCAGCTACTCCCAGGAAGTGGGAGGACCTTTTAAGACACCCAT
TAAGGAAACGCTGCCCATCTCCTCCACCCCGAGCAAATCTGTCCT
CCCCAGAACCCCTGAATCCTGGAGGCTCACGCCCCCAGCCAAAG
TAGGGGGACTGGATTTCAGCCCAGTACAAACCTCCCAGGGTGCC
TCTGACCCCTTGCCTGACCCCCTGGGGCTGATGGATCTCAGCACC
ACTCCCTTGCAAAGTGCTCCCCCCCTTGAATCACCGCAAAGGCTC
CTCAGTTCAGAACCCTTAGACCTCATCTCCGTCCCCTTTGGCAAC
TCTTCTCCCTCAGATATAGACGTCCCCAAGCCAGGCTCCCCGGAG
CCACAGGTTTCTGGCCTTGCAGCCAATCGTTCTCTGACAGAAGGC
CTGGTCCTGGACACAATGAATGACAGCCTCAGCAAGATCCTGCT
GGACATCAGCTTTCCTGGCCTGGACGAGGACCCACTGGGCCCTG
ACAACATCAACTGGTCCCAGTTTATTCCTGAGCTACAGGGTATG
GACTACAAGGATGACGATGACAAGGATTACAAAGACGACGATG
ATAAGGACTATAAGGATGATGACGACAAATGAGCTAGCCTGTGG
AATGTGTGTCAGTTAGGGTGTGGAAAGTCCCCAGGCTCCCCAGC
AGGCAGAAGTATGCAAAGCA

Note: The underlined sites represent the cleavage site and the red lettering indicate the coding region.
